# Supplementary material for: A Structural Comparison of SARS-CoV-2 Main Protease and Animal Coronaviral Main Protease Reveals Species-Specific Ligand Binding and Dimerization Mechanism
Source: Int J Mol Sci. 2022 May 18;23(10):5669. doi: 10.3390/ijms23105669 (PMC9145999; doi:10.3390/ijms23105669)
Supplement: Supplementary file 1 [file ijms-23-05669-s001.zip › ijms-1732844-supplementary.pdf]

Supplementary materials

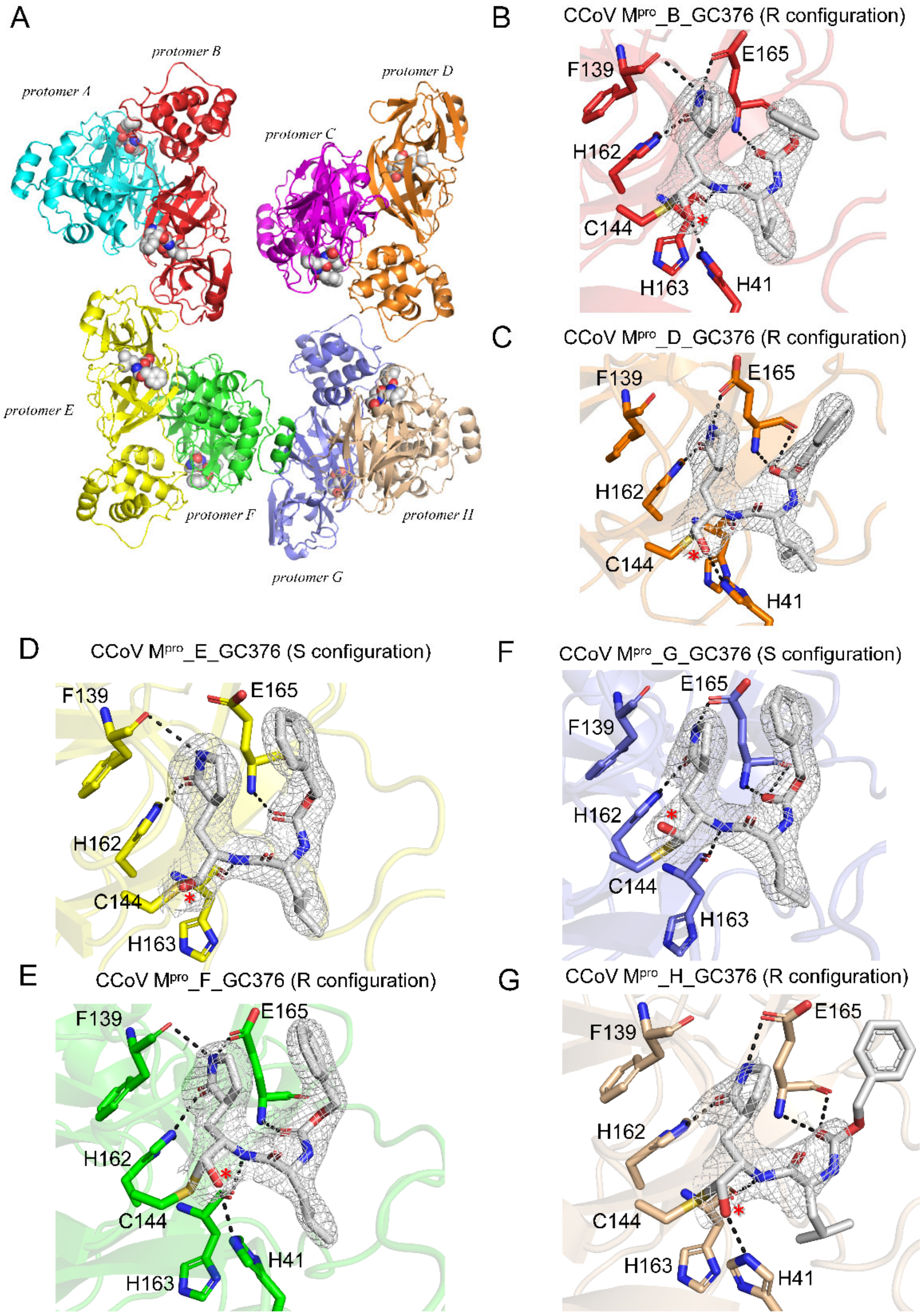

Figure S1. The differences between protomers of CCoV Mpro in the same asymmetric unit. (**A**) The overview of the asymmetric unit of CCoV Mpro complexed with GC376 solved in this study. GC376s are shown in white spheres. (**B-G**) The enlarged views of the substrate-binding pocket from (**B**) protomer B (red), (**C**) protomer D (orange), (**D**) protomer E (yellow), (**E**) protomer F (green), (**F**) protomer G (slate) and (**G**) protomer H (wheat) of CCoV Mpro_GC376 complex. The structures are presented in the same way as Figure 3B-3E. The 2*F*o-*F*c electron density map of GC376s contoured at 1.0 σ are shown in grey meshes.

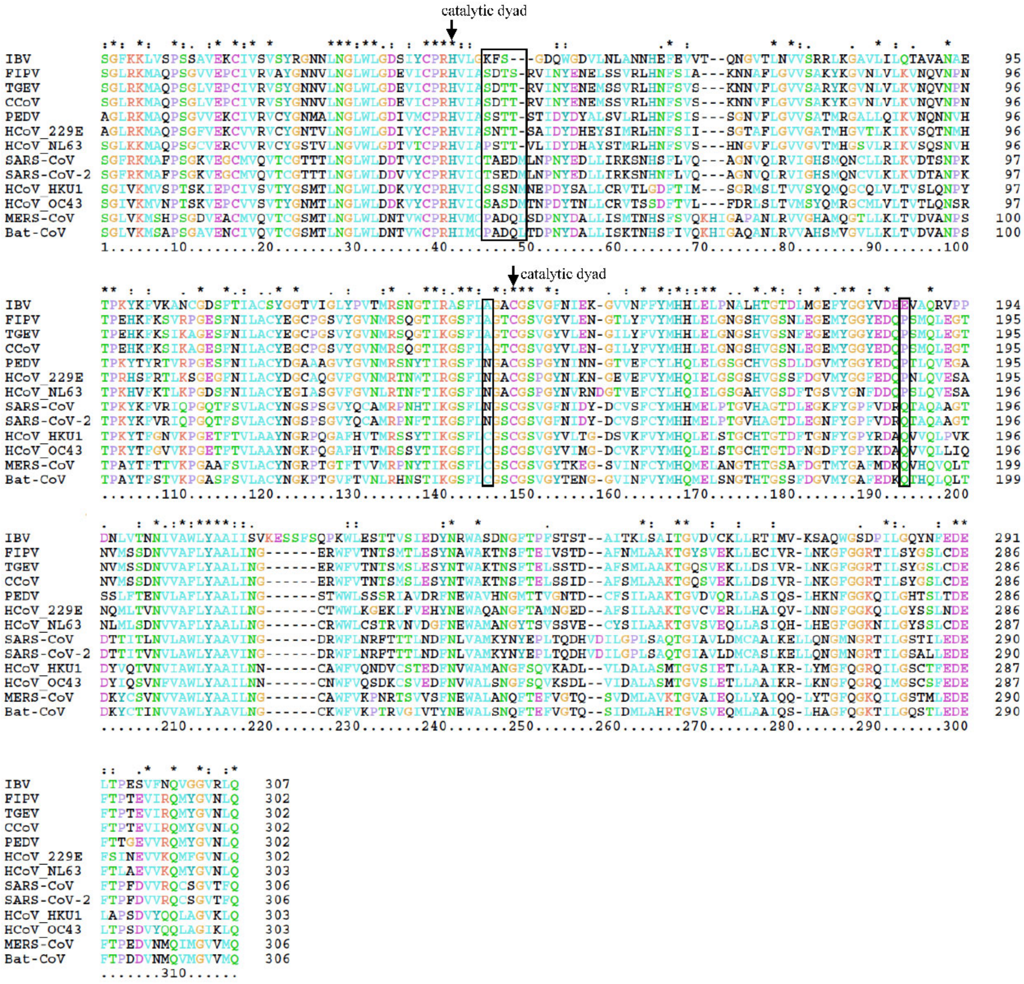

Figure S2. Multiple sequence alignment of animal and human CoV M^{pro}s**.** The conserved catalytic dyad (Cys-His) among all CoV M^{pro}s is indicated by arrows. The three structural features shown in Figure 4 are highlighted by black rectangles. HCoV_229E M^{pro} (GenBank: AGW80947.1, a.a. 2966-3267); HCoV_NL63 M^{pro} (UniProtKB: P0C6U6.1, a.a. 2942-3238); HCoV_HKU1 M^{pro} (UniProtKB: P0C6U3.1, a.a. 3338-3631); HCoV_OC43 M^{pro} (GenBank: AGT51636.1, a.a. 3247 to 3549); MERS-CoV M^{pro} (GenBank: QLD98008.1, a.a. 3248 to 3553); SARS-CoV M^{pro} (GenBank: AFR58684.1, a.a. 3241-3546); SARS-CoV-2 M^{pro} (UniProtKB: P0DTD1, a.a. 3264-3569); Bat-CoV HKU4 M^{pro} (UniProtKB: P0C6F7.1, a.a. 3299-3604); FIPV M^{pro} (UniProtKB: Q98VG9.2, a.a. 2904-3205); CCoV M^{pro} (UniProtKB: P0C6F7.1, a.a. 3299-3604); TGEV M^{pro} (GenBank: AMB66486.1, a.a. 2879-3180); PEDV M^{pro} (UniProtKB: P0C6V6.1, a.a. 2998-3299).

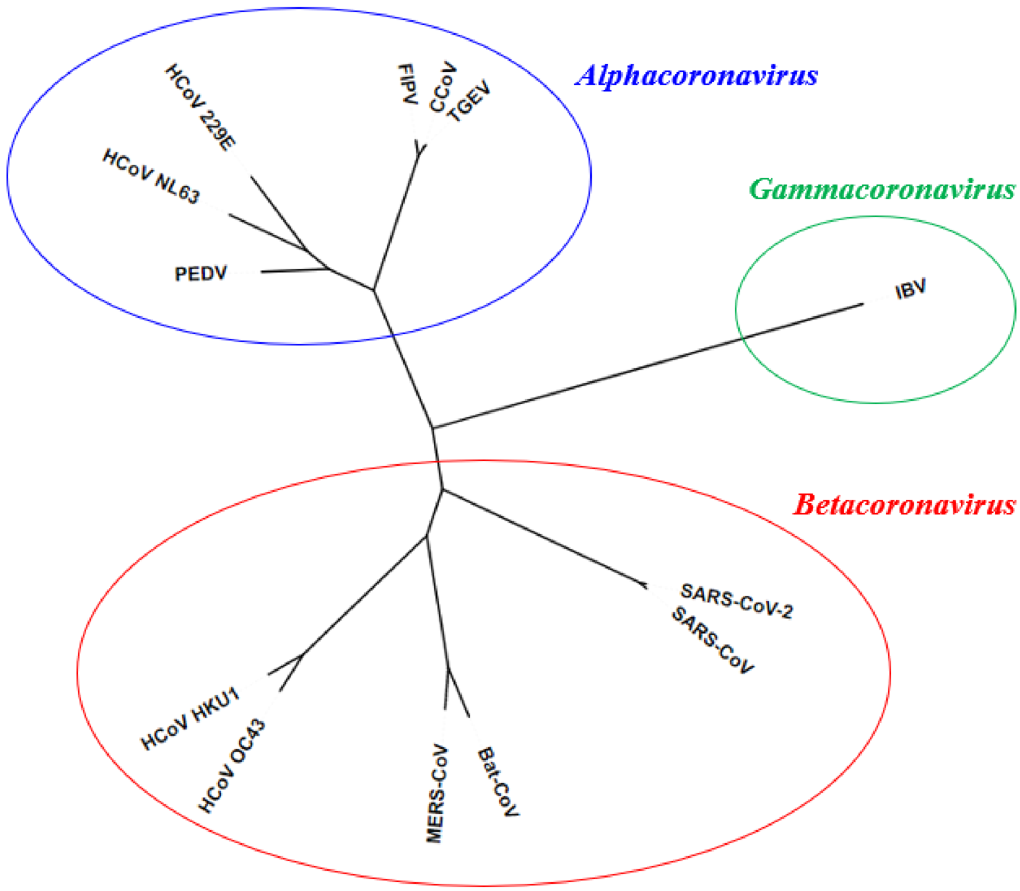

Figure S3. Phylogenetic tree of CoV M^pro^s**.** The source of the sequences of different CoV M^pro^s are identical to those shown in Figure S2. The three different genera of coronaviruses are indicated. .
